# dNTP Supply Gene Expression Patterns after P53 Loss

**DOI:** 10.3390/cancers4041212

**Published:** 2012-11-20

**Authors:** Tomas Radivoyevitch, Yogen Saunthararajah, John Pink, Gina Ferris, Ian Lent, Mark Jackson, Damian Junk, Charles A. Kunos

**Affiliations:** 1 Departments of Epidemiology and Biostatistics, General Medical Sciences (Oncology), and Pathology, Case Western Reserve School of Medicine, Cleveland, OH 44106, USA; E-Mails: jrp16@case.edu (J.P.); ijl4@case.edu (I.L.); mwj7@case.edu (M.J.); djj40@case.edu (D.J.); 2 Department of Translational Hematology & Oncology Research, Taussig Cancer Institute, Cleveland Clinic, 9500 Euclid Ave. R40, Cleveland, OH 44195, USA; E-Mail: saunthy@ccf.org; 3 Department of Radiation Oncology, University Hospitals Case Medical Center and Case Western Reserve School of Medicine, Cleveland, OH 44106, USA; E-Mails: gjf3@case.edu (G.F.); charles.kunos@UHhospitals.org (C.A.K.)

**Keywords:** p53, Li-Fraumeni, ribonucleotide reductase, p53R2, dNTP supply and demand, gene expression, thymidine kinase, deoxyguanosine kinase, deoxycytidine kinase

## Abstract

Loss of the transcription factor p53 implies mRNA losses of target genes such as the p53R2 subunit of human ribonucleotide reductase (RNR). We hypothesized that other genes in the dNTP supply system would compensate for such p53R2 losses and looked for this in our own data and in data of the Gene Expression Omnibus (GEO). We found that the *de novo* dNTP supply system compensates for p53R2 losses with increases in RNR subunit R1, R2, or both. We also found compensatory increases in cytosolic deoxycytidine kinase (dCK) and thymidine kinase 1 (TK1) and in mitochondrial deoxyguanosine kinase (dGK), all of the salvage dNTP supply system; in contrast, the remaining mitochondrial salvage enzyme thymidine kinase 2 (TK2) decreased with p53 loss. Thus, TK2 may be more dedicated to meeting mitochondrial dNTP demands than dGK which may be more obligated to assist cytosolic dNTP supply in meeting nuclear DNA dNTP demands.

## 1. Introduction

Resistance to cytotoxic anticancer therapies often arise by losses in p53 [1]. Whether p53 loss-related mechanisms of resistance introduce new cancer cell vulnerabilities exploitable by anticancer therapies is not known. Because p53 drives transcription of the p53R2 subunit of ribonucleotide reductase (RNR) [2], p53 loss may lower *de novo* deoxyribonucleoside triphosphate (dNTP) supply needed for DNA repair after DNA damaging anti-cancer therapies. Characterizing how dNTP supply compensates for p53 (and thus p53R2) losses is therefore of interest.

**Figure 1:**
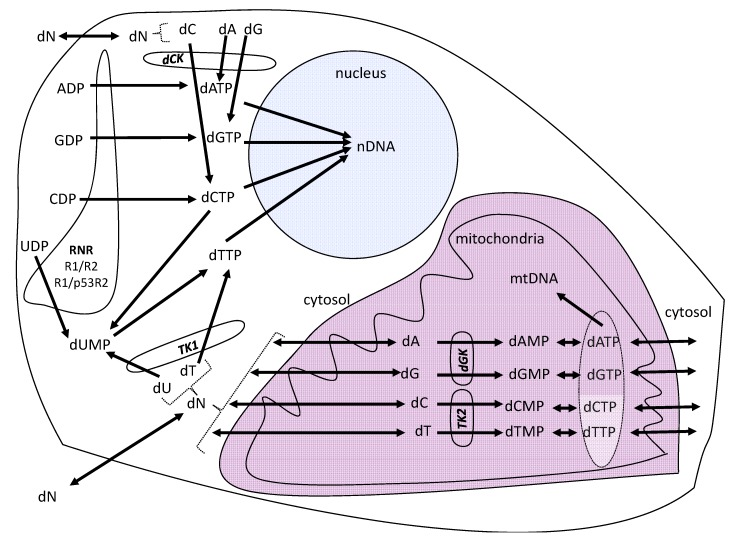
dNTP supply and demand. Enzymes of the dNTP supply system of interest are shown in bold. They include RNR, dCK and TK1 in the cytosol and dGK and TK2 in the mitochondria. Other important dNTP supply enzymes (e.g., nucletotidases) have been suppressed for clarity.

The dNTP supply system (Figure 1) is comprised of a *de novo* pathway that is rate-limited by RNR formed as either an R1/R2 or R1/p53R2 complex, and a salvage pathway that is rate-limited by the cytosolic enzymes deoxycytidine kinase (dCK) and thymidine kinase 1 (TK1) and by the mitochondrial enzymes deoxyguanosine kinase (dGK) and thymidine kinase 2 (TK2). The *de novo* and salvage pathways are coordinated such that the total dNTP fluxes supplied equal the total demanded by nuclear and mitochondrial DNA replication and repair.

The wiring diagram in Figure 1 predicts that cells could potentially compensate for p53R2 loss mediated decreases in *de novo* dNTP supply by: (1) increasing deoxynucleoside (dN) salvage enzyme levels; (2) elevating levels of RNR subunit R1 such that very low levels of p53R2 available in cells have an increased chance of partnering with R1 to form functional R1/p53R2 complexes while simultaneously creating a surplus of S/G2 phase R1/R2 complexes that might create a surplus of dNTPs that can spill over into other cell cycle phases; or (3) boosting RNR subunit R2 levels in S/G2 phase as an alternative means of achieving dNTP spillovers. Determining the extent to which each of these possibilities occurs may assist researchers in understanding how treatment resistant cancer cells defective in p53 differ from normal cells. For example, if compensation for unmet dNTP demand induced by loss of p53R2 occurs predominantly by salvage enzyme compensation, deoxynucleoside analogs such as gemcitabine [3] and decitabine (DAC) [4] may be optimal for treating cancers of such cells; DAC therapy would then have the additional advantage of promoting cell cycle exit by p53-independent differentiation mechanisms [5]. And if p53R2 loss is compensated for mostly by elevation in R1 or R2 levels, or if resistance to dC analogs arises by mutations in dCK [6,7], a drug such as triapine that inhibits p53R2 and R2 [8] without dCK traversal might be a better therapeutic strategy. In this manner, advancing our understanding of dNTP supply adjustments after p53 losses could lead to improved, personalized cancer therapies.

In this study we examine the dNTP supply system response to p53 loss by analyzing gene expression data from human mammary epithelial cells (HMEC) minimally transformed by stably knocking down p16, with and without p53 also knocked down, and data from the gene expression omnibus (GEO) [9] that includes: (a) normal human stromal breast tissue cells of Li-Fraumeni and healthy donors [10], (b) breast cancer cells that are p53 wild-type or mutated [11], and (c) KB cancer cells with p53R2 knocked down by p53R2 siRNA [12].

## 2. Results and Discussion

### 2.1. Comparison of HMEC with and without p53

**Figure 2:**
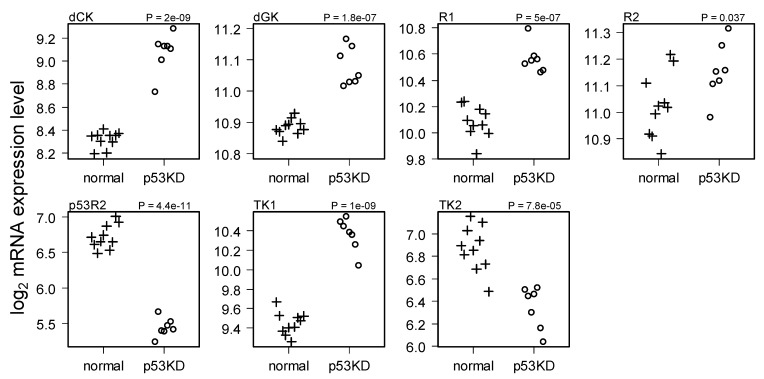
Steady state HMEC dNTP supply compensation for p53 and thus p53R2 loss. dCK, dGK, R1, R2 and TK1 increase while TK2 decreases. *P* values are for t-test comparisons of cells with (+) *versus* without (o) p53, *i.e*., normal *versus* p53KD (p53 knocked down). Probe sets on Affymetrix U219 chips were averaged after conversion to log base 2 units. On the y-axis “10” represents 2^10^ = 1024 (logarithms stabilize variances). Robust multi-chip analysis [14,15] was used throughout to achieve quantile normalization across chips. Left to right random jittering within groups was used here and below to improve visualization of individual data points.

**Figure 3:**
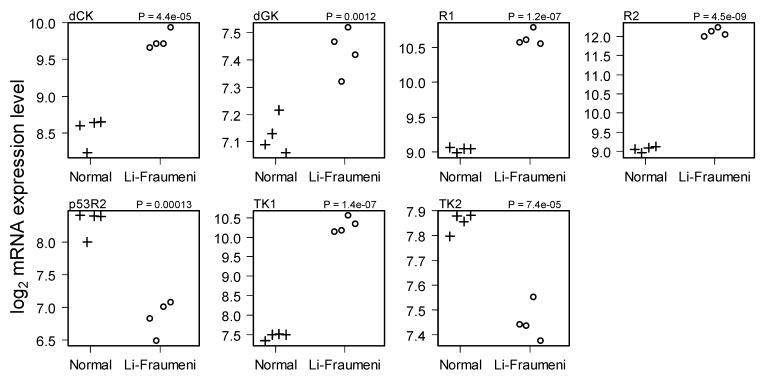
Stromal breast tissue data of Herbert *et al.* [10] supports Figure 2. Data is from GEO accession GSE23994. This is Affymetrix U133 2.0 plus data. *P* values are for t-test comparisons of cells with (+) *versus* without (o) p53.

**Figure 4:**
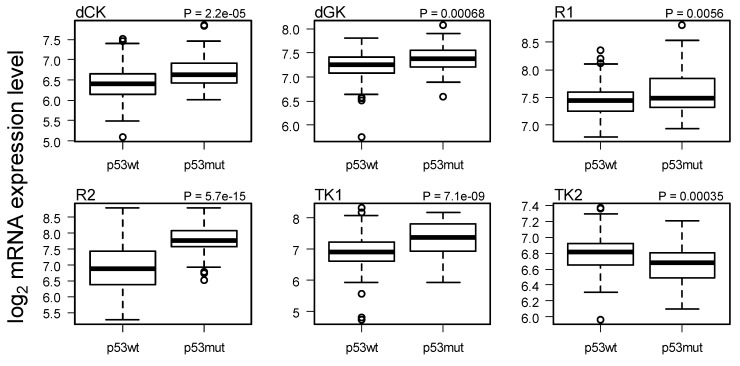
Breast cancer tissue microarray data of Miller *et al*. [11]. T-test *p* values are shown.

**Figure 5:**
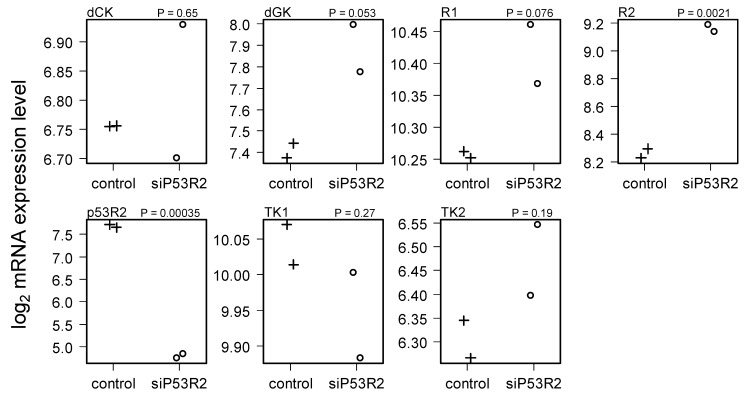
Cancer cell gene expression responses to p53R2 knock-down. Despite only four measurements, t-test comparisons of cells with (+) *versus* without (o) p53R2 were used to capture strong pairing in R2 and p53R2.

**Figure S1:**
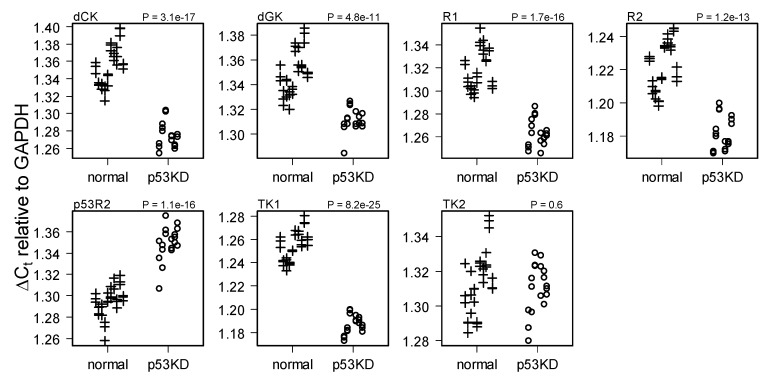
RT-PCR results corresponding to Figure 2. On the y-axis ΔC_t_ indicates the number of cycles needed to detect the target minus the number needed to detect GAPDH, *i.e*., larger values indicate lower mRNA levels. Consistent with Figure 2, in p53 knock down (p53KD) cells p53R2 levels are lower and dCK, dGK, R1, R2 and TK1 levels are higher. TK2 decreases that were expected based on array data in Figure 2 were not confirmed.

HMECs with or without p53 were exposed to ionizing radiation (IR, 2 Gy) with or without 5 μM triapine (3-aminopyridine-2-carboxaldehyde thiosemicarbazone, 3-AP) for 6 hours and followed for 24 hours. We were interested in 3-AP because it has been combined successfully with IR to treat human cancers demonstrating functional p53 loss [13]. The impact of 3-AP and IR were, however, minimal, except at 24 hours. Measurements across the first 6 hours were thus pooled as equivalent to pretreatment replicates (Figure 2) that, as steady state measurements, could then be compared to other published steady state data (Figure 3, Figure 4 and Figure 5). Our results (Figure 2) show that p53 loss caused a decrease in p53R2; since p53 is controlled by protein degradation, its transcript is generally uninformative, but transcripts downstream of the p53 transcription factor can serve as reporter surrogates, and p53R2 is ideal for this because it also relevant to our system. Figure 2 also shows increases in the cytosolic dNTP supply enzymes dCK, TK1, R2, and R1, and a split in the mitochondrial salvage enzymes, with dGK increasing but TK2 decreasing. One interpretation of this is that dCK and TK1 are adequate for deoxypyrimidine salvage needed for nuclear dNTP demands, but in meeting this demand, an unwanted excess is created with respect to mitochondrial dNTP demands such that TK2 must now decrease to annihilate this perturbation. Meanwhile, dGK increases are consistent with dCK needing assistance to produce purine dNTP supply fluxes demanded by both nuclear and mitochondrial DNA. All of these changes were confirmed by quantitative RT-PCR (Figure S1), save the decrease in TK2 where Figure S1 instead supports no change; 2 of 3 GEO results below (Figure 3, Figure 4 and Figure 5) support a TK2 decrease, and none support an increase, so TK2 decreases are supported when all of the data are viewed collectively.

### 2.2. Comparison of Normal and Li-Fraumeni Gene Expression

We searched GEO for relevant data to see if our observed pattern could be substantiated. In this section we describe the Li-Fraumeni patient data of Herbert *et al.* [10], in subsequent sections we explore other data. Focusing here on only the breast stromal tissue samples of this dataset, on grounds that it is less dependent on monthly hormone variations than breast epithelium, our results are shown in Figure 3. Here, four independent samples were obtained from each of two subjects: one an age-matched healthy subject, the other a p53-loss verified Li-Fraumeni subject. The consistency between this data and our own data is striking. Statistically significant increases in R1, R2, TK1, dCK, and dGK, and decreases in p53R2 and TK2, were observed, as in our own data, though with different magnitudes, e.g., in R2.

### 2.3. Comparison of Gene Expression in Breast Cancer with and without p53 Mutated

In GEO accession GSE3494, 58 p53-mutated cancer patients were distinguished from 193 p53 wild-type subjects [11] and characterized using Affymetrix U133A chips that do not contain a probe set for p53R2. The pattern (Figure 4), namely, increases in dCK, TK1, R1, R2 and dGK, and a decrease in TK2, was the same as that observed in Figure 2 and Figure 3.

### 2.4. Gene Expression in Cells Treated with siP53R2

Finally, we explored GEO dataset GSE25238 wherein p53R2 was suppressed directly using siRNA [12]. We found (Figure 5) that R2 increased significantly in response to p53R2 loss, even with only two samples per group (*i.e*., a total of four measurements). Furthermore, though none of the other genes studied changed significantly (save the internal positive control of p53R2), dGK and R1 bordered on significance in directions consistent with Figure 2, Figure 3 and Figure 4. Speculating, this may suggest that whereas these genes and R2 responded to a lack of dNTP supply (caused by a lack of p53R2), dCK and TK1 compensation in Figure 2, Figure 3 and Figure 4 may in part be due to direct p53 loss effects not mediated by p53R2, *i.e*., p53 may have other effects on dNTP supply outside of those caused by p53R2 loss.

## 3. Experimental Section

### 3.1. Cell Lines and Culture Conditions

Normal pre-stasis HMEC, a kind gift from Martha Stampfer (Lawrence Berkeley National Laboratories, Berkeley, CA, USA), were originally isolated from discarded tissue acquired following reduction mammoplasty (specimen 48R batch T). HMEC and their derivatives were grown in a humidified environment at 37 °C with 5% CO_2_ in M87A medium with oxytocin as previously described [16].

### 3.2. Viral Vectors and Infection

The retroviral vector SINhygro-shp16 (shp16) was provided by Scott Lowe (Cold Spring Harbor Laboratory, Cold Spring Harbor, NY, USA). Retroviruses were produced as previously described [17]. Briefly, retroviral vectors were transfected into Phoenix-Ampho cells together with a packaging plasmid encoding the MLV gag, pol, and env genes. The lentiviral vector pLV-shp53-bleo encoding short-hairpin-RNA targeting p53 (shp53) [18] was packaged in 293T cells using the second-generation packaging constructs pCMV-dR8.74 and pMD2G, kind gifts from Didier Trono (University of Geneva, Geneva, Switzerland). Viral supernatant media containing virus was collected in M87A medium for 24 hours, filtered with a 0.22 µm filter, supplemented with 4 μg/mL polybrene, and added to HMEC for infection overnight (18 hours). Uninfected cells were removed by selection with puromycin (1 µg/mL) or hygromycin (200 µg/mL).

### 3.3. RNA Isolation

Total RNA was isolated from cell lysates using an RNeasy® mini kit (Qiagen, Valencia, CA, USA) according to manufacturer protocols. RNA quality was tested by denaturing formaldehyde 1% agarose gel electrophoresis. RNA was quantified using a Nanodrop ND-1000 spectrophotometer (Thermo Scientific, Wilmington, DE, USA). RNA processing and microarray analysis was performed in the Gene Expression and Genotyping Facility of the Case Comprehensive Cancer Center. Total RNA was converted to hybridization cocktail using the Affymetrix 3' IVT Express Kit. PolyA RNA controls were included throughout the process to monitor the target labeling process. In addition, a mixture of biotinylated, fragmented cRNA bacterial hybridization controls were also included to serve as indicators of hybridization efficiency. Hybridization cocktails were loaded into 36 wells of a 96-well plate. Samples were arrayed in a random manner among the 4 × 8 layout of the wells. Samples were then hybridized to the Affymetrix Human Genome U219 peg arrays, washed, stained, scanned and digital images were archived using the Affymetrix GeneTitan MC automated system.

### 3.4. Data Analysis

Cel files for all 36 of our own samples and for data downloaded from GEO [9] were processed and analyzed using Bioconductor [19]. Briefly, cel files were converted into Bioconductor ExpressionSet objects using the robust multichip analysis (RMA) method of the R package affy [14,15]. The ExpressionSet was then analyzed using the R packages limma [20] and hgu219.db of Bioconductor [21]. RMA includes probe-level quantile normalization across all of the cel files in a data set (e.g., 36 in our data).

## 4. Conclusions

**Figure S2:**
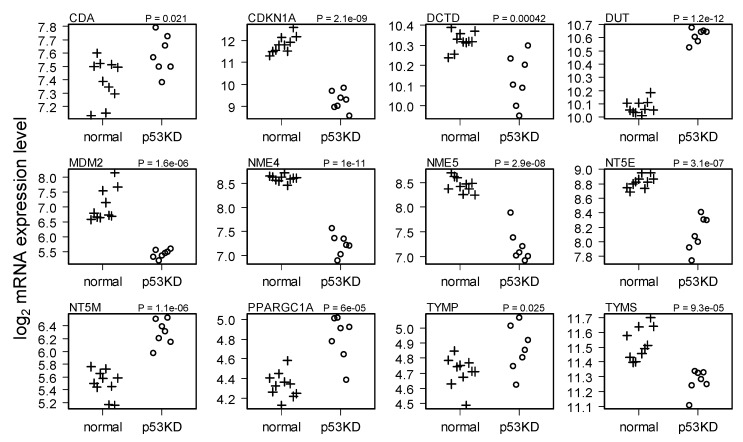
HMEC data.

**Figure S3:**
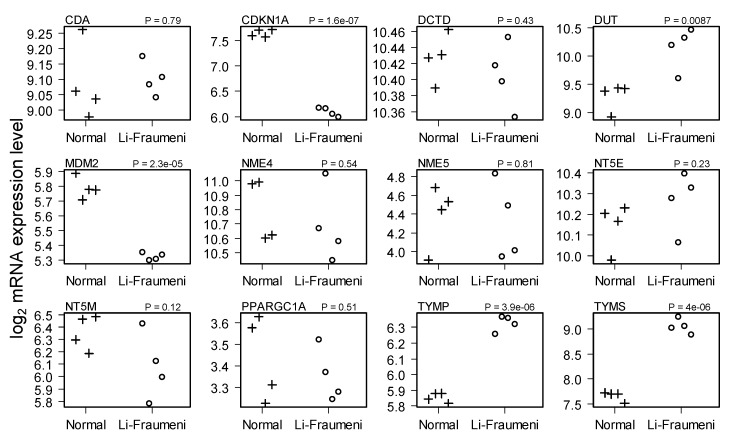
TYMS here contradicts TYMS in Figure S2. (Herbert *et al*. stromal breast tissue).

**Figure S4:**
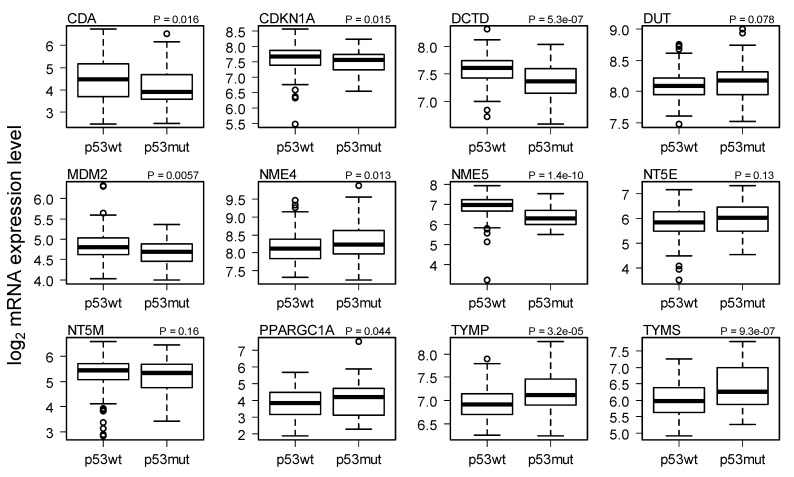
TYMP and TYMS here are consistent with Figure S3. (Miller *et al*. Breast Cancer Patients).

**Figure S5:**
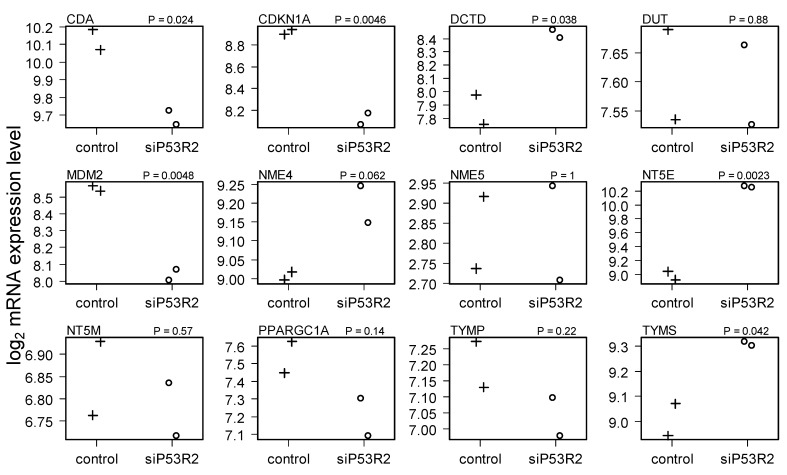
CDA and DCTD here contradict their levels in Figure S2. (Zhang *et al*. p53R2 KD).

Our results suggest that after p53 loss, R1, R2, dCK, TK1 and dGK increase, and TK2 decreases. We speculate that genes that increased prioritize nuclear dNTP demands, and by meeting them, overproduce deoxypyrimidines with respect to mitochondrial demands. In contrast, we speculate that TK2 is more dedicated to meeting mitochondrial dNTP demands, so it compensates for what it sees as cytosolic dNTP concentrations that are too high by decreasing its level so that within mitochondria, dNTP supply matches dNTP demand. In this picture, relative to TK2, dGK has broader obligations to both nuclear and mitochondrial dNTP demands, consistent with dCK’s affinity for dG being much less than dGK’s affinity for dG [23] and dCK thus being in need of help from dGK for purine dNTP supply control. That such compensations are incapable of overcoming complete p53R2 loss is evidenced by mitochondrial DNA depletion in muscles of children with p53R2 defects [22]. However, as our findings are only at the mRNA level, they await confirmation at the protein level and thus remain highly speculative. Also, other enzymes of dNTP supply were explored (supplemental Figure S2, Figure S3, Figure S4 and Figure S5, corresponding to Figure 2, Figure 3, Figure 4 and Figure 5) but they did not reveal a consistent pattern, e.g., TYMS was elevated in all of the datasets save our own. Figure S2, Figure S3, Figure S4 and Figure S5 did, however, confirm p53 losses, as MDM2 and CDKN1A, viewed as reporters of p53, did decrease with losses of p53.

Because pyrimidine and purine nucleoside analogue oncotherapies require cellular uptake by salvage pathways and are antagonized by *de novo* pathway activity [24], alterations in these pathways induced by p53 loss may be an important element in the therapeutic index of these drugs, particularly for the pyrimidine analogue decitabine that can induce cell cycle exit independent of p53 [5].

A logical next step is to develop mathematical models of dNTP supply [25] that incorporate our findings, and use the models for control system design [26,27], *i.e*., to optimize nucleoside analogue cancer therapies [28] using methods proposed for chronic myeloid leukemia [29,30,31]. We believe that it is through such applications that systems biology will have its greatest impact on cancer therapy.

## Supplementary Materials

Confirmation by PCR. In Figure S1 we confirm six of seven results in Figure 2, the exception being for TK2. The details of the methods used are as follows. All quantitative PCR procedures were performed in the Case Comprehensive Cancer Center Gene Expression and Genotyping Core Facility. RNA was quantified using a nanodrop-1000 spectrophotometer (Nanodrop Industries). Archive cDNA was made for all samples by means of an RT reaction using ABI high-Capacity cDNA archive kit using 3 μg of total RNA as starting material in a 100 μL reverse transcription reaction in an ABI 9700 PCR unit. 384-well plates were set up to accommodate triplicate reactions for all assays. The plate runs showed good amplification and good replication within assays. An endogenous control assay was used to control for RNA loading. Assays (RRM1, RRM2, p53R2, TK1, TK2, dGK and dCK) were purchased from ABI and set up in accordance with the manufacturer’s instructions for a 40 cycle run. Spectral data, gathered during the PCR run, were converted into numerical data using ABI SDS 2.3 proprietary software. Results are presented as relative fold changes versus a designated calibrator sample (GAPDH). The software algorithm used the delta-delta Ct method to calculate RQ (relative quantitation) values.

Other dNTP supply related array data genes. In Figure S2, Figure S3, Figure S4 and Figure S5, corresponding to Figure 2, Figure 3, Figure 4 and Figure 5, the dNTP supply related genes, cytidine deaminase (CDA), dCMP deaminase (DCTD), dUTPase (DUT), NDP kinases (NME4, NME5), nucleotidases (NT5E, NT5M), dTMP synthetase (TYMS), dT phosphorylase (TYMP) and the mitochondrial biogenesis gene PPARGC1A, show no consistent pattern. In contrast, similar to p53R2, CDKN1A and MDM2 consistently report p53-protein losses as expected.
